# Impulsiveness and Cognitive Patterns. Understanding the Perfectionistic Responses in Spanish Competitive Junior Athletes

**DOI:** 10.3389/fpsyg.2019.01605

**Published:** 2019-07-16

**Authors:** Juan González-Hernández, Concepción Capilla Díaz, Manuel Gómez-López

**Affiliations:** ^1^Departamento de Personalidad, Evaluación y Tratamiento Psicológico, Facultad de Psicología, Universidad de Granada, Granada, Spain; ^2^Faculty of Health Sciences, University of Granada, Granada, Spain; ^3^Faculty of Sport Sciences, University of Murcia, San Javier, Spain

**Keywords:** emotional stability, functional perfectionism, young athletes, sport competition, impulsiveness

## Abstract

**Introduction:**

High performance sport requires that the athletes maintain a constant intensity and control of their personal resources, as well as a balance between self-regulation and performance. Likely, such requirements involve the influence of their beliefs regarding the tasks to be performed to improve the confidence in their own resources to face the competition. Theoretical arguments provide new insights for understanding multidimensional perfectionism and its relationships with other variables such as affective experiences, among others. In this study, perfectionism was conceptualized as a “stable personality disposition,” whereas the impulsiveness components were defined as “representing psychological mechanisms (or processes)” underlying the relationships between perfectionism and athletic experiences.

**Aim:**

This study aims to establish and show profiles of perfectionist beliefs and impulsive responses according to sport modality and the relationships between all these variables. Team athletes were expected to show more functional resources than those in combat or endurance sports.

**Methods:**

The psychological responses of 487 athletes (273 boys; 214 girls) practicing high-performance sport were examined. A non-randomized, cross-sectional design was used. Self-reports were used to measure impulsiveness, perfectionism and competence self-perceptions.

**Results:**

Athletes with functional responses of impulsivity and perfectionism showed higher perceived self-competence. Athletes with more reflective thoughts, more careful planning and generally less sensitive to rewards and behaviors were more self-regulated and planned (functional impulsivity) and showed more moderate relationships between the most dysfunctional perfectionist beliefs and self-competence. In addition, perfectionism seems to be useful to the striver athletes that want to be the best, and they are focused on and committed to future goals and performance and self-improvement. It is important for coaches and athletes to understand how the processes of self-regulation (impulsivity) and self-knowledge (perfectionism) could be formed to try to offer better opportunities for building psychological resources that enhance high-performance mental abilities.

## Introduction

The study of impulsiveness, considered the internal force that generates the activation of a person, has traditionally been oriented toward the loss of control and difficulties in managing impulses derived from such activation (Bari and Robbins, 2013). The beliefs and skills related to how to achieve either impulse self-regulation or control, according to what the behavior or performance is, determine the interpretation of these as either a barrier or a reinforcement, with the person being unable to restrain signs of anger, anxiety or fear (in the case of interpretation as a barrier), or attachment or sensation stimulations (in the case of interpretation as a reinforcement) (Corr, 2004; Stoeber and Corr, 2015). In this sense, athletes must be aware of this ambivalence to be able to educate and train themselves and, in this way, to try to manage situations that pose an internal conflict in an assertive way (Laborde et al., 2018).

Cognitively, impulsiveness is characterized by the absence of reflexive control and the anticipation of posterior consequences, which leads to the making of mistakes in performance situations (Dalley et al., 2011; Smith et al., 2016). Emotionally, impulsiveness is characterized by a low tolerance for frustration, inability to delay reinforcements, little resistance to temptation and few control resources for regulating the impulses to quickly respond (Bridgett et al., 2015; Guinote, 2017), which is in opposition to the qualitative reactions of anger, happiness, anxiety, euphoria, or fear (neurotic response) (Cook et al., 2018). Socially, impulsiveness has been linked with socialization difficulties (Van Stekelenburg and Klandermans, 2017), low empathy (Baldner et al., 2015), prosociality (Do et al., 2017), dependence relations (Odacı and Çelik, 2016), aggressiveness (Johnson and Carver, 2016), and manipulation (Salekin, 2016).

In sports, functional impulsiveness requires the presence of high concentration (Kovářová and Kovář, 2010; Gustavson et al., 2014) and adequate self-regulation skills to achieve greater efficiency and speed in decision making or emotional expression (González-Hernández and Garcés de Los Fayos, 2014; Laborde and Allen, 2016; Cook et al., 2018). For example, for a taekwondo fighter to make the final attack to close the fight, for a golfer to decide the most appropriate blow on the next hole, or for a tennis player to deal with a disputed point, they need to maintain a high level of activation, which they have previously developed and processed as automatism in their execution of psychological training.

Perfectionism is considered both a one-dimensional and multi-dimensional trait of our personality, represented by beliefs of a self-demand for excellence and the inclination to set high standards for performance, followed by a hypercritical self-evaluation and high concern focused on errors (to avoid making them) (Frost et al., 1990). In recent years, different authors have defined perfectionism by setting the emphasis on interpersonal facets and social rules (socially prescribed perfectionism) or the internal aspects of individuals oriented toward achieving goals and aims (self-oriented perfectionism) (Flett and Hewitt, 2014; Madigan et al., 2015; Vicent et al., 2016).

By studying perfectionism in the field of sports, it is necessary to discern between perfectionist efforts and perfectionist concerns (Stoeber, 2014). On the one hand, perfectionist efforts (with an adaptive effect) are positively related to competitive self-confidence, positive emotions, hope for success, task orientation, and objectives focused on performance and mastery in both training situations and competition (Jowett et al., 2016). González-Hernández and González Reyes (2017) understand that the construction of perfectionist beliefs allows athletes to adapt to demands, allowing them to have less vulnerability to anxiety (Moshier et al., 2016). In fact, perfectionistic efforts has been negatively related to competitive anxiety and fear of failure. On the other hand, perfectionist concerns are related to emotions that can be considered negative (e.g., cognitive, competitive and pre-competitive anxiety, low self-esteem, fear of failure and avoidance, and practice and mastery of skills) (Koivula et al., 2002; Correia et al., 2015). Correia et al. (2015) consider that perfectionist concerns can have a predictive effect on the fear of failure and, therefore, a dysfunctional effect on the athlete both in competition and in training.

Furthermore, regarding the components of evolutionary and cognitive processes, which are in constant interaction with their contexts, processes such as perfectionism or impulsiveness have been characterized on base of functional or dysfunctional characteristics of humans. Perfectionism is functional when belief schemes have high performance standards in combination with an uncritical evaluation of them (Lo and Abbott, 2013; Mautz et al., 2017). Perfectionism is dysfunctional when high personal standards are associated with an excessively critical self-evaluation (Flett and Hewitt, 2006, González-Hernández et al., 2018). Impulsiveness has been described as functional through self-regulated emotions and behaviors (Boone et al., 2014; Macfarlane et al., 2016). In contrast, dysfunctional impulsiveness processes have been described when there is low emotional quality (De Silva et al., 2016; MacKillop et al., 2016), lack of impulse control (Smillie and Jackson, 2006; Donovan et al., 2014; Harden et al., 2017) and disruptive behavior (Renee-Renda et al., 2018).

Conceptualizations of perfectionism and impulsiveness in sports practice (Stoeber, 2014; Stoeber and Madigan, 2016; Stoeber and Gaudreau, 2017) are associated with tendencies to excessively assess the level of concern for execution errors (e.g., “*The execution must to be perfect; any small failure is a failure*.”), self-evaluation (e.g., “*Have I done it well or have I missed something?*”), feelings of uncertainty (e.g., “*What will happen now?*”), and parental and external expectations, as well as referring to contextual elements that are not very controllable and that do not depend on oneself (e.g., “*What will others think?*”) or by giving too much importance to precision, order and organization (e.g., “*This is life for me; here, I cannot fail.*”). In essence, these are perfectionist concerns that are related to, on the one hand, high levels of fear of failure, stress, depression, anxiety and illness and, on the other hand, low levels of confidence in sports performance and satisfaction with tasks. In contrast, and mainly focused on self-security, perfectionism has also been associated with the capacity to make efforts and, at the same time, has been related to indicators of subjective well-being and psychological adjustment (Sirois and Molnar, 2016), greater motivation for participation in training and self-determined behavior in competitions, greater mastery of and orientation in tasks (showing a preference for difficult tasks) (Stoeber and Gaudreau, 2017), high self-confidence, better relationships among peers, and greater self-esteem and better coping strategies in difficult situations (Stoeber and Corr, 2015; González-Hernández and González Reyes, 2017).

The immediacy with which one lives the sport practice, which is excessively related to competition along with the rivalries that are constructed during the competition, and the excess of activation are sources of dysfunctional behaviors (Stoeber et al., 2009; see Table 1). Nevertheless, impulsiveness is an important factor in motor performance interference in open-skill sports modalities (e.g., *basketball, hockey, or volleyball*) that experience constant changes in the environment (e.g., *alterations in opponent positioning or changes in partial results*) (Poltavski and Biberdorf, 2015; Ellingson et al., 2019) and where the player is forced to inhibit pre-planned responses, anticipate actions and coordinate corporal segments based on the complex and dynamic flow of sensorial information (Lage et al., 2011).

**TABLE 1:** Functional and Dysfunctional behaviors in athletes.

**Functional impulsivity-perfectionism**	**Dysfunctional impulsivity-perfectionism**
Athlete looks for exciting experiences and assumes more risky goals.	Athlete shows a low tolerance for frustration and boredom.
Athlete acts before thinking independently of the situation-problem.	Athlete is disorganized and almost never plans activities.
Athlete is very creative, although many of his proposals are sketches that need to be polished.	Athlete is very forgetful and / or because of lack of foresight.
Athlete is clear about which objectives to direct his efforts.	Athlete changes from one activity to another very frequently.
Athlete is motivated and acts with determination.	Athlete is unable to keep calm to make decisions about their actions.
Athlete improves their efficiency in basic resources for sports performance (concentration, memory, reaction time, decision making, etc.).	Athlete requires a lot of supervision to avoid problems.
Athlete is able to understand when to need the help of others.	Athlete gets angry easily or maintain conflicts with figures in their environment.
Athlete has problems for acting inappropriately.	Athlete demands the help or asks others not to fail.

Finally, considering the variability of such psychological responses as perfectionism and impulsivity and taking into account the characteristics that may be established between their functionality and dysfunctionality, the aims of this study are as follows: (a) to show the indicators of perfectionism and impulsivity in young athletes in the stage of sports technification and belonging to Spanish sports federations; (b) to reflect the linear relationships between them; and (c) to identify the differences in these relationships, according to the sports modality. To do this, a non-random, predictive and cross-sectional study has been designed with the hope of fulfilling the hypothesis that athletes who show a more functional impulsivity are associated with patterns of functional perfectionism and that those who show a more dysfunctional impulsivity are likely to show more patterns of dysfunctionality in terms of perfectionist beliefs. Those who practice more team sports are expected to show more functional responses than those who practice combat or endurance sports.

## Materials and Methods

### Sample

The sample was composed of young Spanish athletes (*N* = 487), from two technification levels (under 23 years and under 19 years), who resided at the specialized centers of different sports federations and others sport clubs in different parts of Spain. Their age range was between 16 and 23 years old (Mage = 18.76; *SD* = 3.15). The average competition experience was 7.46 years (*SD* = 3.62), and the average weekly training session was 15.86 h/week (*SD* = 3.05). Regarding gender, the sample included 273 boys (50.60%) and 214 girls (43.94%). Sports modalities were grouped into three categories (see Table 2): sports combat (taekwondo, judo, karate, and boxing), team sports (soccer, basketball, hockey, volleyball, and handball), and endurance sports (open water swimming, triathlon, and BMX).

**TABLE 2:** Socio-sport, dispersion and descriptors data of participants.

	**General**		**Gender**			**Sport technification level**		
		
	**M_age_** **(DE)**	**As-K**	**Boys**	**Girls**	**As-K**	**U19_years_**	**U23_years_**	**As-K**
Combat sports (*n* = 128)	16.73 (4.17)	0.33 (−0.31)	76 (59.37%)	52 (40.63%)	0.42 (−0.23)	74 (57.81%)	54 (42.18%)	0.26 (0.31)
Team sports (*n* = 244)	19.84 (4.82)	0.42 (0.26)	134 (54.92%)	110 (45.08%)	0.34 (0.29)	147 (60.25%)	97 (39.75%)	0.32 (−0.20)
Endurance sports (*n* = 115)	19.23 (3.85)	0.28 (−0.13)	63 (54.78%)	52 (45.22%)	0.33 (−0.27)	43 (37.39%)	62 (53.91%)	0.37 (0.18)

*As, Asymmetry; K, Kurtosis. U19_*years*_, Under 19 years old (Junior category); U23_*years*_, Under 23 years old (Promises category).*

### Instruments

#### Perfectionism

The Multidimensional Perfectionism Scale was used (FMPS, Frost et al., 1990, adapted to the Spanish population by Carrasco et al., 2010). This scale is formed by 35 items and consists of four dimensions of the first order (personal norms, organization, concern about mistakes, and doubt related to actions) and two of the second order (functional and dysfunctional). The response options for each of the items cover a range of 5 points on a Likert scale, with 1 meaning “*in total disagreement*” and 5 meaning “*completely agree*.” The internal reliability of the questionnaire is high, with α = 0.87.

#### Impulsiveness

The Barrat Impulsiveness Scale for adolescents was applied (BIS-11-A; Spanish adaptation and validation for Martínez-Loredo et al., 2015). This version kept up the 30 items of the original scale, linguistically adapted to adjust for coherence in sport contexts. Participants report, on a Likert scale, the frequency of different behaviors, with 1 being “*rarely or never*,” 2 being “*occasionally*,” 3 being “*often*,” and 4 being “*almost always* or *always*.” It is distributed in two subscales: general impulsiveness (e.g., “*I am happy-go-lucky*” *or* “*I change hobbies and sports*”) and non-planned impulsiveness (e.g., “*I plan what I have to do*” *or* “*I like to think about complex problems*”). The scale is distributed in 30 items, the response range was between 30 and 120, and the internal consistency was α = 0.86.

### Procedure

First, the research was forwarded to and approved by the Human Research Ethics Committee of the University of Granada. The heads of sports centers (different federations and sports clubs in Spain) were informed about the reasons for and content of the investigation, the guidelines to be followed and requests for the pertinent permits. After consent was given, the protocol was established as follows: (a) there was a meeting with coaches (sometimes, the managers are the coaches), (b) an informed consent document was facilitated through email, in person or by smartphone for each parent/tutor in the case of minors or directly in the case of adults, until the corresponding authorizations were obtained.

Finally, to protect and maintain ethical norms, the researchers explained the confidentiality, anonymity of data management and privacy, according to the American Psychological Association’s ethical guidelines (World Medical Association, 2013). A member of the research team was always present to explain, resolve doubts about the answers, and maintain the scientific rigor for the proper application of the instruments.

### Data Analysis

Different descriptive analyses (frequencies, central tendency, and dispersion) of socio-sport variables (gender, sport, and sports level) were carried out. Differential analysis was performed for gender (discriminant analysis) and sport modality (ANOVA with an interpretation of Bonferroni test and factor f for effect size), and the Kolgomorov–Smirnov test and Cronbach’s alpha were used to assess compliance with the normality and statistical reliability, respectively. The possibility of making type I and type II errors in the hypothesis analysis was considered. Pearson correlation analysis (r) was performed to assess the linear relationship of the dimensions studied, and a radar graphic was created to show the description and differences of the profiles of the associated qualities and attributes, according to the practiced sports modality. The statistical package program IBM SPSS 23.0 was used.

## Results

Regarding the aim of showing the differential data about the direct relations between perfectionism beliefs and the impulsiveness response, we computed correlations of the impulsiveness subscales with second-order dimensions of perfectionism (functional and dysfunctional) in each one of the three sports modalities established (Table 3). It can be considered that functional perfectionism points to a direct, positive and significant relation with non-planned impulsiveness in all sports modalities studied while maintaining an inverse and significant relation with general impulsiveness, except for team sports, which did not show significant relationships.

**TABLE 3:** Correlations between perfectionism beliefs and impulsive response in young Spanish athletes.

		**Combat sports**		**Team sports**		**Endurance sports**	
	**K-S**	**GIM**	**NPIM**	**GIM**	**NPIM**	**GIM**	**NPIM**
Adaptive perfectionism	0.16	−0.59^∗∗^	0.53^*^	–	0.34^∗∗^	−0.62^*^	−0.35^*^
Desadaptive perfectionism	0.21	0.24^*^	−0.52^∗∗^	0.44^*^	−0.56^∗∗^	0.52^*^	−0.49^∗∗^

^*^Significant < 0.05; ^∗∗^Significant < 0.01.

Otherwise, dysfunctional perfectionism showed direct, positive and significant relationships with general impulsiveness but maintained inverse and significant relationships with non-planned impulsiveness in all sports modalities studied.

At the same time, “general impulsiveness” indicated positive correlations with concerns about mistakes (*r* = 0.47; *p* < 0.03), doubts related to actions (*r* = 0.52; *p* < 0.00), personal standards (*r* = 0.52; *p* < 0.01), and inverse relationships with the organization (*r* = 0.75; *p* < 0.00). Non-planned impulsiveness was mainly related in a positive way to the organization (*r* = 0.60; *p* < 0.00) and inversely to parental expectations (*r* = 0.63; *p* < 0.01), parental criticism (*r* = 0.57; *p* < 0.00), and doubts related to actions (*r* = 0.49; *p* < 0.00).

**FIGURE 1:**
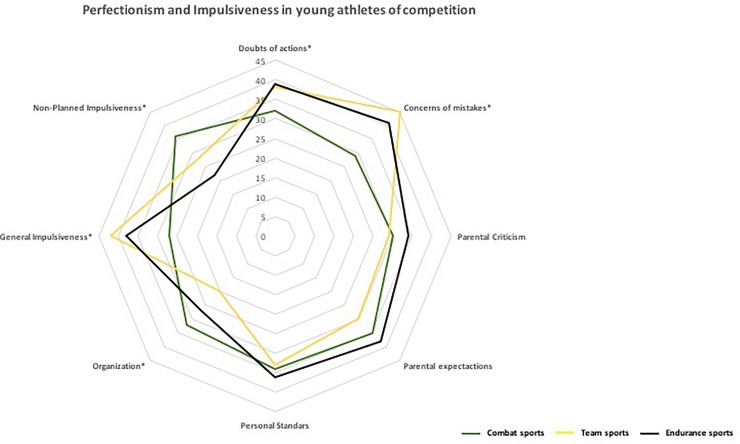
Perfectionism and impulsiveness in young athletes of competition.

Univariate analyses according to gender indicated adequate discriminants (λ = 0.89; X2 = 33.57; *p* < 0.01), indicating significant functions of maladaptive perfectionism and general impulsivity in favor of boys and significant functions of non-planned impulsivity in favor of girls (78.3%). A comparison of the three sports modalities studied (Figure 1) showed that the means are lower for organization in team sports than in both combat sports (ß = 0.16; *p* < 0.00; *f* = 0.28) and endurance sports (ß = 0.19; *p* < 0.01; *f* = 0.32), and the means are higher in concerns about mistakes (*p* < 0.00), general impulsiveness (ß = 0.21; *p* < 0.00; *f* = 0.27) and doubts related to actions (ß = 0.15; *p* < 0.01; *f* = 0.34) with respect to combat sports but not endurance sports. Thus, endurance sports had higher means for general impulsiveness (ß = 0.18; *p* < 0.02; *f* = 0.26) than combat sports but not team sports. Combat sports had higher means for non-planned impulsiveness than both endurance sports (ß = 0.16; *p* < 0.00; *f* = 0.25) and team sports (ß = 0.17; *p* < 0.00; *f* = 0.28) and higher means for organization than both endurance sports (ß = 0.20; *p* < 0.01; *f* = 0.33) and team sports (ß = 0.17; *p* < 0.01; *f* = 0.36). Finally, combat sports showed lower scores in doubts related to actions than team sports (ß = 0.19; *p* < 0.02; *f* = 0.24) and endurance sports (ß = 0.18; *p* < 0.02; *f* = 0.30) and lower scores in concern about mistakes than both team sports (ß = 0.18; *p* < 0.00; *f* = 0.28) and endurance sports (ß = 0.20; *p* < 0.00; *f* = 0.24).

There were no significant differences between the three sports modalities in terms of parental criticism and personal standards.

## Discussion

For the purpose of analyzing perfectionism and impulsiveness scores in a sample of young athletes in competition, the present work describes how athletes of different sports modalities show different profiles and functional resources related to their psychological response.

First, inverse relationships were shown between functional perfectionism dimensions and general impulsiveness in athletes of combat and endurance sports but not of team sports. However, all modalities showed positive relationships with non-planned impulsiveness, as proposed by the first hypothesis.

These data indicate that athletes build different functional resources (such as perfectionism and impulsivity) based on the needs of each sport modality (e.g., combat sports have a philosophy in which athletes must obtain many self-regulation skills and important technical gestures). All of this makes the athlete work and train to learn how to plan, how to be more self-disciplined, how to focus on the present and how to pay less attention to other distracting stimuli (Williams et al., 2015), so such athletes tend to have high standards and minimal concerns (Suarez-Cadenas et al., 2016).

Although it could be considered that combat can lead to aggressive or impulsive behaviors, athletes of combat sports showed lower scores for general impulsiveness, as similar studies have shown (Parthi, 2013; Gronek et al., 2015). Other studies have indicated that practitioners of endurance sports (e.g., BMX, open water swimming, and triathlon) dispose of higher impulsiveness indicators (Monasterio, 2013; Hřebícková et al., 2014). Moreover, our sample showed inverse correlation scores between functional perfectionism and general impulsiveness. In this way, functional perfectionistic beliefs are considered worthy resources for managing situations in sports or other performance modalities (Stoeber et al., 2009; Gucciardi et al., 2012; Gouttebarge et al., 2015a). It is very likely that ordered thought and self-regulation sensations are the best strategies for psychological responses under pressure (Laborde et al., 2018). In team sports, this relationship has also been found in studies on rugby players (Gherghişan, 2015), volleyball players (Palmateer, 2016), and football players (Gouttebarge et al., 2015b). Thus, dysfunctional perfectionism responses showed positive relationships with dysfunctional impulsiveness and inverse relationships with functional impulsiveness in all sports modalities.

Some studies have also linked impulsiveness to positive outcomes such as lower reaction time (Congdon et al., 2010), high sensation seeking (Corr and Krupić, 2017), creativity (Kipper et al., 2010) and adventure behaviors (Quilty et al., 2014).

With reference to the second hypothesis, there were substantial differences among modalities. Combat sports showed that their athletes develop more functional resources than do those of the other modalities. Team and endurance sports athletes had the highest scores in dysfunctional resources. The literature on sports psychology, focused on disorders or alterations in behaviors, has shown that for training in this kind of sport, it helps to have high scores in perfectionism and low self-regulation (Hřebícková et al., 2014; Gouttebarge et al., 2015b; Nixdorf et al., 2016; Prinz et al., 2016).

In contrast, personal standards and parental criticism did not show differences in any of the sports modalities, which made us think that athletes, independent of their disciplines, understand sports competition in a similar way, with high self-demands to reach their athletic aims independent of the sport and modality (Dunn et al., 2006).

Similarly, although paternal figures influence perfectionistic beliefs by their high criticism and expectations (Appleton et al., 2010; Hill et al., 2014), such criticism did not show significant differences in the present study.

## Conclusion

By analyzing the interactions between aspects related to sports success, this study will provide valuable information for coaches and athletes to enhance personal resources and psychological skills toward competition, which will allow them to make adjustments during training seasons.

For an athlete, striving to achieve the best result for success, fulfillment and improvement is similar to “his/her law” or his/her faith. However, the disappointment of achieving something less than “the best” often causes the athlete shameful and negative feelings. Researchers have examined the positive and negative sides of this double-edged sword. Perfectionist efforts (also described as “adaptive or functional perfectionism”) help athletes to gain pleasure from their efforts. However, they also allow them to accept limitations and setbacks, even criticism. Building such functionally perfectionist beliefs in athletes or coaches directly influences their motivation, emotions, balance, and self-confidence. In fact, perfectionism is often seen as either a reinforcement in sports and performance contexts, (e.g., security in actions).

Athletes who describe striving for the best, focusing on future goals and performances, benefit from their perfectionism and are more likely to set goals. As a consequence, they commit themselves to working hard and self-improvement, generating a “perfect” and focused result in their thoughts, self-regulations and feelings. Self-regulation skills, planning skills, emotional management skills or the definition of expectations should be personal resources in psychological training, from which athletes can train and/or restructure their perfectionist beliefs and impulsive responses. Thus, a better understanding of competitive situations and sports planning (e.g., macro cycles, micro cycles, and competitions), the management of exaggerated pressure maintained over time or communication and empathic skills with their athletes will help coaches build better training environments and connections with their athletes. This will facilitate the mental growth that these athletes need to guide them in their competitive and non-competitive moments.

The findings present a number of limitations focused on the difficulties in data collection and the need to acquire permissions for entry into the changing rooms of clubs and sports institutions. Sports contexts are environments with high possibilities to show the importance of the connections between temperament and character resources under pressure responses in athletes. In this study, only two variables were considered, but we can reflect on more (e.g., narcissism and self-regulation). Therefore, new proposals will consider designs with more variables (e.g., predictive models or models combining three variables at different levels) and longitudinal research designs that can help to show the continuity or changes in the psychological response of athletes in their competition seasons, categories or personal circumstances (e.g., injuries or sport transition).

## Author Contributions

JG-H developed the methodological proposal and data analysis, realized the literature review, and wrote the part of the theoretical frame. MG-L described the “Conclusion” and “References” sections. CCD collaborated in data analysis and results redaction.

## Conflict of Interest Statement

The authors declare that the research was conducted in the absence of any commercial or financial relationships that could be construed as a potential conflict of interest.
